# Analysis of Global RNA Synthesis at the Single Cell Level following Hypoxia

**DOI:** 10.3791/51420

**Published:** 2014-05-13

**Authors:** John Biddlestone, Jimena Druker, Alena Shmakova, Gus Ferguson, Jason R. Swedlow, Sonia Rocha

**Affiliations:** ^1^Centre for Gene Regulation and Expression, College of Life Sciences, University of Dundee, UK

**Keywords:** Cellular Biology, Issue 87, Cancer, RNA synthesis, Hypoxia, Microscopy, Click-iT, Open Microscopy Environment, OMERO

## Abstract

Hypoxia or lowering of the oxygen availability is involved in many physiological and pathological processes. At the molecular level, cells initiate a particular transcriptional program in order to mount an appropriate and coordinated cellular response. The cell possesses several oxygen sensor enzymes that require molecular oxygen as cofactor for their activity. These range from prolyl-hydroxylases to histone demethylases. The majority of studies analyzing cellular responses to hypoxia are based on cellular populations and average studies, and as such single cell analysis of hypoxic cells are seldom performed. Here we describe a method of analysis of global RNA synthesis at the single cell level in hypoxia by using Click-iT RNA imaging kits in an oxygen controlled workstation, followed by microscopy analysis and quantification.  Using cancer cells exposed to hypoxia for different lengths of time, RNA is labeled and measured in each cell. This analysis allows the visualization of temporal and cell-to-cell changes in global RNA synthesis following hypoxic stress.

**Figure:**
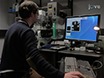


## Introduction

Hypoxia (low oxygen tensions) occurs when the normal oxygen supply to a tissue is disturbed. Environmental oxygen is both a nutrient and a signaling molecule, providing important cues for many cell types. Changes in environmental oxygen are sensed by a group of dioxygenases that control the activity of an essential transcription factor family known as the Hypoxia Inducible Factors (HIFs). The HIFs are composed of two subunits, α and β. There are three known isoforms of HIF-α (1, 2, and 3) and multiple splice variants of HIF-1β. HIF-1β is constitutively expressed and not regulated by environmental oxygen levels^1^. The HIF-α family members are dynamically regulated by a class of prolyl-hydroxylases (PHDs) and the Factor Inhibiting HIF (FIH); both of which require oxygen as a co-factor to catalyze the hydroxylation of HIF-α^2,3^. In normoxia the HIF-α family members are hydroxylated and tagged for proteosomal degradation by the E3-Ligase, von Hippel Lindau (vHL). In hypoxia the PHDs and FIH are inactive or have a reduced activity. The HIF-α isoforms become stabilized, form a heterodimer with HIF-1β, and effect the transcription of genes that provide the cellular response to the hypoxic environment (**Figure 1A**)^4^.

Current techniques for RNA analysis focus on the quantification of averaged values across a given cell population. Cells respond to a hypoxic stimulus by initiating the transcription of a myriad of genes that allow them to adapt to their hostile environment^5^. However, hypoxia often exists as a gradient, and cells in a hypoxic environment are not subject to a uniform hypoxic stimulus. We describe an implementation of the Click-iT RNA imaging kits in an oxygen controlled workstation to examine global RNA synthesis at the single cell level in hypoxia.

The RNA imaging kit uses an alkyne-modified nucleoside, 5-ethynyl uridine (EU) and chemoselective ligation to enable detection of global RNA synthesis temporally and spatially in cells and tissues^6^.  Briefly, cells are treated with hypoxia and cultured in the presence of EU. They are then fixed and permeabilized and EU incorporation into nascent RNA is detected by chemoselective ligation of EU with an azide containing dye. A typical workflow for this reaction is shown in **Figure 1B**. We used the RNA imaging kit to examine RNA synthesis at the single cell level that resulted from treatment with hypoxia.

The small size of the alkyne tag enables efficient incorporation of the modified nucleoside into RNA specifically. The chemoselective ligation or 'click' reaction is highly efficient, fast and specific^7-10^. All of the reaction components are bioinert and the reaction requires no extreme temperatures or solvents. The click reaction negates the requirement for conventional radiolabelling and allows direct visualization of the results since the output is light. In addition, the detection molecule can easily penetrate complex samples allowing for multiplex analysis including antibodies for the detection of RNA-interactive proteins. This RNA imaging assay is compatible with organic dyes including Alexa Fluor and fluorescein (FITC).

We measured the change in RNA synthesis following treatment of our cells using an implementation of the open microscopy environment for remote objects (OMERO). OMERO is open-source software, available at http://openmicroscopy.org/ . This microscope image visualization and analysis software enables access to, and use of a wide range of biological data, including the management of multidimensional, heterogeneous datasets. The client application allows remote visualization and analysis of complex biological image data^11^; we used it to quantify the visual changes in global and single cell RNA synthesis. These data and the steps required to analyze our RNA imaging experiment using this microscope image visualization and analysis software are shown below.

We looked at changes in global RNA synthesis following the treatment of human osteosarcoma (U2OS) cells with hypoxia for up to 24 hr. In all conditions, we detected cell-to-cell variation in the level of RNA production. Short times of hypoxia exposure did not result in significant changes to the level of nascent RNA in cells. However, exposure to 24 hr of hypoxia resulted in a significant increase in the amount of RNA produced in cells. Most of the cellular responses to hypoxia are measured following prolonged periods of exposure, such as 4 to 24 hr. However, some mechanisms are put in place much earlier, for example; NF-κB activation occurs within 5-15 min of hypoxia exposure^12^. Investigating shorter hypoxia exposure times is therefore warranted and could detract from more complicated responses such as cell cycle and apoptosis.

## Protocol

### 1. Cell Culture and Treatment

1. Gather the reagents and equipment listed in **Table 1** to culture human osteosarcoma (U2OS) cells. Prepare all reagents and conduct all tissue culture techniques in the laminar air flow hood to ensure sterility
2. Prepare the culture medium as follows: Add 50 ml FCS, 5 ml L-glutamine and 5 ml penicillin/streptomycin to the DMEM to achieve final concentrations of 10% (FCS), 2 mM (L-glutamine), 50 U/ml (penicillin) and 50 U/ml (streptomycin). Warm the culture medium at 37 °C in a water bath.
3. Prepare the cells for treatment:
  1. Sterilize several coverslips in 70% ethanol, allow to air dry and place one on the bottom of six 3.5 cm plates.
  2. Immerse the coverslips in 2 ml warmed complete DMEM (37 °C), pressing down with the coverslip forceps to ensure that they remain adhered to the bottom of the wells.
  3. Detach the U2OS cells from their tissue culture plate by washing once with 3 ml PBS, applying 4 ml warmed 0.05% trypsin-EDTA (1%) and incubating at 37 °C for 7 min.
  4. Resuspend cells in 6ml warmed complete DMEM to inactivate the trypsin-EDTA and count using a hemocytometer.
  5. Plate 2 x 10^5^ cells to each of the 3.5 cm plates prepared in point 1.3.1. Gently rock the plate to ensure an even cell distribution. Allow the cells to adhere overnight prior to treatment.
4. Treat the cells by placing four 3.5 cm plates into the hypoxia workstation and leave the other two 3.5 cm plates in the incubator at 37 °C in normal oxygen conditions. Be ready to commence the RNA labelling assay under treated conditions **(in the hypoxia chamber for hypoxia treated cells) **1 hr before the hypoxia treatment ends.
5. Expose cells to hypoxia for 1 hr 15 min, 1 hr 30 min, 2 hr, and 24 hr. These time points are flexible and user-determined.
6. Use a normoxic 3.5 cm plate as the positive control and a normoxic 3.5 cm plate treated with Actinomycin D (10 µg/ml final concentration) for 4 hr as the negative control. Actinomycin D is an inhibitor of global RNA synthesis.

### Cautions:

Hoechst 33342 is a known mutagen. DMSO is known to facilitate the entry of organic molecules into tissues. NaOH is corrosive. HCl is corrosive. Paraformaldehyde is highly toxic to all animals and can cause death in humans. Triton X-100 can cause skin irritation following direct contact. Actinomycin D is toxic when in contact with skin or swallowed. Take appropriate precautions when handling all hazardous chemicals.

### 2. Click-iT Assay

1. Gather the following reagents that are required in addition to the Click-iT assay kit: phosphate buffered saline (PBS) pH 7.2 - 7.6, 3.7% paraformaldehyde (PFA) in PBS, 1% Triton X-100 in PBS, deionized water (dH_2_O), dimethyl sulfoxide (DMSO), 10 M sodium hydroxide (NaOH), and 37% hydrochloric acid (HCl); Optional - pH indicator strips.
2. Prepare a fresh PFA stock solution before performing the Click-iT reaction as follows:
  1. Put 1.85 g PFA, 3.5 ml dH_2_O and 10 µl of10 M NaOH into a 50 ml Falcon tube. Boil 300 - 400 ml of H_2_O in a large beaker in the microwave to make a water bath and stand this in a fume hood.
  2. Place the 50 ml Falcon into the water bath and gently agitate for 10 min, periodically releasing the lid until the PFA has dissolved.
  3. Syringe the PFA solution through a 0.2 µm filter into another 50 ml Falcon tube, resulting in a 37% solution.
  4. Dilute this 10x by adding PBS and bring the pH to 6.8 by adding about 12 µl of concentrated HCl. Option: Confirm the correct pH with the indicator strips in the fume hood.
  5. Use immediately or store overnight at 4 °C.
3. Prepare the stock solutions of the RNA imaging kit as follows:
  1. Add 373 µl dH_2_O to component A resulting in a stock solution of 100 mM of EU. Store at -20 °C for up to one month.
  2. Add 85 µl of DMSO to component B (Alexa Fluor 594) and mix by pipetting or vortexing. Store both at -20 °C for up to one year.
  3. Add 2 ml dH_2_O to component E to create a 10X solution of the RNA imaging kit reaction buffer additive. Store at -20 °C for up to one year, discard when solution develops a brown color.
4. Labeling of Cells with EU: Prepare a 2X working solution of EU from the 100 mM stock prepared in step 2.3 in prewarmed complete medium (37 °C). **Perform the remainder of this step and step 2.5 in the hypoxia chamber for cells treated with hypoxia. **Dilute to 1X by adding an equal volume of this 2X working solution to the media containing cells. Incubate for 1 hr under treatment cell culture conditions.
5. Cell Fixation: Wash each well once with PBS and add 1 ml of the 3.7% PFA stock prepared in step 2.2 above under treatment conditions. Incubate for 15 min under treatment conditions. **The samples treated with hypoxia can be removed from the hypoxia chamber at this point**. Remove the fixative and wash each well once with PBS (**Take care to dispose of PFA waste responsibly**). Perform the next steps at room temperature.
6. Permeabilization: Remove the wash solution and add 1 ml of 1% Triton X-100 in PBS to each well. Incubate for 15 min at room temperature. Remove the permeabilization buffer and wash each well once with PBS.
7. Labeled RNA Detection:
  1. Prepare a fresh 1X working solution of RNA imaging kit reaction buffer additive by diluting the 10X solution (prepared in step 2.3) 1:10 in dH_2_O.
  2. Prepare the RNA imaging kit reaction cocktail according to **Table 2** for use immediately after preparation.
  3. Remove the wash solution and add 500 µl of RNA imaging kit reaction cocktail to each sample. Incubate for 30 min at room temperature, **protect from light**.
  4. Remove the RNA imaging kit reaction cocktail and wash once with 1 ml of RNA imaging kit reaction rinse buffer (component F), then remove the RNA imaging kit reaction rinse buffer.
8. Optional multiplex reaction: Perform additional antibody labeling of samples at this point according to manufacturer's recommendations.
9. DNA Staining: Wash samples with PBS then remove the wash solution. Dilute the Hoechst 33342 (Component G) 1:1,000 in PBS. Add 1 ml of diluted Hoechst 33342 to each well and incubate for 15 min at room temperature, **protect from light**. Remove the Hoechst 33342 solution and wash the cells twice with PBS. Remove the wash solution and proceed to light microscopy.

### 3. Light Microscopy

1. Mount Coverslips:
  1. Place 10 µl of mounting medium on the center of a standard microscope slide.
  2. Place the coverslip with cell-side down on the center of the microscope slide to cover the aliquot of mounting medium. Take care to avoid the generation of bubbles in the mounting medium as this will obscure subsequent image acquisition.
  3. Stick the coverslip to the microscope slide by applying clear nail varnish to its perimeter. Allow to dry for 5 min at room temperature. **Protect from light**. Samples may be stored at -20 °C following mounting.
2. Image acquisition: Acquire images using a wide-field microscope capable of fluorescence detection. Approximate fluorescence/excitation emission maxima for the Alexa Fluor dye and Hoechst 33342 bound to DNA are shown in **Table 3**.
3. Perform Imaging using a 40X/1.30 NA oil immersion lens and capture images with a cooled CCD camera.

### 4. Data Analysis

1. Deconvolve images using image processing software (see Materials table) prior to importing to the OMERO client. Normalize rendering settings for all images in the same experiment in the microscope image visualization and analysis software by applying the rendering settings from control normoxic cells to all other conditions.
2. Use the Region Of Interest (ROI) tool in the microscope image visualization and analysis software (see Materials table) to select nuclei from each image. Obtain mean intensities for each ROI and calculate the average and standard deviation for each condition by choosing the intensity analysis option, including a number between 30-50 cells.
3. Construct Scatter plots using a data graphing software (see Materials table) by plotting all individual mean intensity values obtained in step 4.2 to reflect variability between cells under each condition. Alternatively construct bar graphs to represent average mean intensity plus standard deviation of nascent RNA.

## Representative Results

A schematic of the RNA imaging kit reaction is shown in **Figure 1B**. We used this reaction to quantitatively determine total RNA synthesis in U2OS cells following treatment with hypoxia on both an individual (single cell) and global level (population of cells). The cells were treated with hypoxia and cultured in the presence of EU. They were then fixed and permeabilized and EU incorporation was detected by chemoselective ligation of EU with an azide containing dye. This 'click' reaction results in a fluorescence emission that can be detected and quantified using fluorescence light microscopy. **Figure 2A** shows a typical result from this experiment. Fluorescently labeled cells are pseudocolored red and the image has been opened in the open microscopy environment for remote objects (OMERO). This microscope image visualization and analysis software has a built-in ROI tool that can be used to select individual sub-cellular structures and quantitatively analyze image intensity within the chosen region. A typical example of the data output following the use of this tool in the client program can be seen in **Figure 2B**; individual cell intensities are shown alongside a representative image from the experiment that serves as a visual check to ensure data validity.

The individual cell fluorescence intensities can be plotted as a scatter chart to show the distribution of the individual cellular intensities within the treated cell population. An example of this type of graph is seen in **Figure 2C**. Plotting intensity data in this way is useful because it allows a rapid comparison of treatment results and clearly demonstrates the significant heterogeneity within a treatment cell population that is otherwise obscured using standard data representation. The response heterogeneity is interesting in this experiment because although a uniform hypoxic stimulus was applied to the cells, it is evident that every cell from the same monolayer population responds differently to this stimulus.

**Figure 2D** depicts the overall result from our experiment. Fluorescence intensities from all cells within a treatment population were averaged and then plotted in this bar chart. A student's t-test has been used to calculate the statistical probability of each treatment group differing significantly from the control. Interestingly, these data show that there is a statistically significant increase in global RNA synthesis over time when U2OS cells are treated with hypoxia (at times greater than 1 hr 30 min. *p < 0.01; **p < 0.05 and ***p < 0.001 and n > 30). These data show that following longer periods of hypoxia treatment, the cell focusses its efforts on the production of RNA even in this hostile hypoxic environment. Actinomycin D treatment completely ablates the fluorescence intensity as expected.


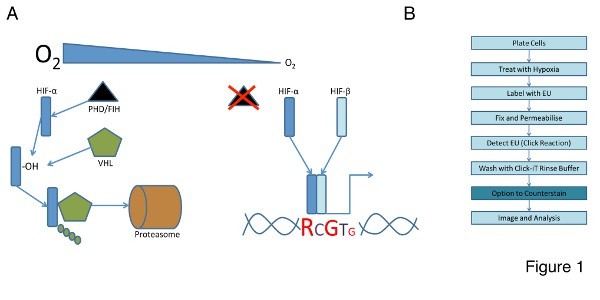
**Figure 1. A.** The HIF system responds to changes in cellular oxygen. In this simplified schematic hypoxia stabilizes the HIF heterodimer by preventing HIF-α hydroxylation (-OH) by the prolyl hydroxylase enzymes (PHDs) and its subsequent polyubquitination by von-Hippel Lindau protein (vHL). HIF stabilization results in the activation of myriad genes that help the cell respond to cellular hypoxia. **B.** RNA synthesis can be detected using the Click-IT assay; cells are labelled with EU after treatment the Click reaction detects RNA synthesis which can be quantified by fluorescence intensity and light microscopy. Please click here to view a larger version of this figure.


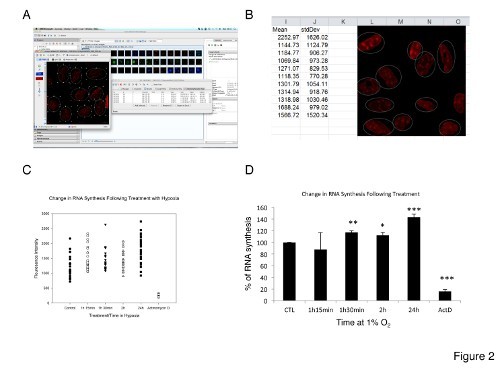
**Figure 2. A.** Screenshot from OMERO to show typical microscopic output following RNA labelling. This microscope image visualization and analysis software can quickly extract intensity data on an individual cell level using its inbuilt ROI macro. A screenshot of the output from this command is shown in **B.** Global RNA synthesis can be inferred from individual cell intensity, the range of which can be seen in scatter plot **C.** A formal representation of these aggregate data can be seen **D.** *p < 0.01; **p < 0.05 and ***p < 0.001 and n > 30. Click here to view larger image.

**Table:** 

**Cell Culture Reagents**
Phosphate buffered saline (PBS) pH 7.2 - 7.6
Dulbecco's Modified Eagles Medium (DMEM)
Fetal calf serum (FCS) filter sterilized
Penicillin/streptomycin
L-glutamine
0.05% Trypsin-EDTA (1%)
70% ethanol
Actinomycin D (1 μg/μl stock)
**Cell Culture Equipment**
Sterile Pasteur Pipettes
A range of cell culture-treated plastic ware suitable for the maintenance and treatment of cells in culture
Water bath set at 37 °C
Laminar air flow hood
Incubator set at 37 °C, 5% CO_2_
Hypoxia Workstation set at 37 °C, 5% CO_2_, 1% O_2_
Glass coverslips
precision forceps
Hemocytometer

**Table 1. Reagents and equipment required for culturing of U2OS cells.**

**Table:** 

**Reaction Components**	**Number of Coverslips**
1	2	4	5	6	10
Click-iT RNA reaction buffer (Component C)	428 μl	856 μl	1.7 ml	2.1 ml	2.58 ml	4.3 ml
CuSO_4_ (Component D)	20 μl	40 μl	80 μl	100 μl	120 μl	200 μl
Alexa Fluor Azide (Prepared in Step 2.3)	1.8 μl	3.7 μl	7.4 μl	9.3 μl	11.28 μl	18.8 μl
Click-iT reaction buffer additive (Prepared in step 2.3)	50 μl	100 μl	200 μl	250 μl	300 μl	500 μl
Approximate Total Volume	500 μl	1 ml	2 ml	2.5 ml	1.5 ml	5 ml

**Table 2. RNA imaging kit reaction cocktails:** Reference table detailing ratio of master mix components for RNA imaging kit reaction according to the number of coverslips to be stained.

**Table:** 

**Fluorophore**	**Excitation (nm)**	**Emission (nm)**
Alexa Fluor 488	495	519
Hoechst 33342, bound to DNA	350	461

**Table 3. Fluorescence/excitation emission maxima:** Approximate fluorescence/excitation emission maximal for Alexa Fluor dye and Hoechst 33342 bound to DNA.

## Discussion

We have described our use of an RNA imaging kit to examine the effects of hypoxia on RNA synthesis in osteosarcoma (U2OS) cells. This technique is relatively straightforward and provides data about global transcription at a single cell level. We modified the conventional protocol for this kit to incorporate labeling in a hypoxia chamber. To our knowledge this is the first report of the use of this technique to measure changes in RNA synthesis in hypoxia. The RNA imaging kit we used is suitable for experimentation in hypoxia since it requires no radioactive isotopes and is technically compatible with the limitations of working in a controlled atmosphere workstation. Common problems with this technique concern efficient fixation and labeling of the sample. It is extremely important that the PFA used to fix the sample is fresh and the wash steps that follow the 'click' reaction are performed as described to ensure low background staining of the sample. If background fluorescence becomes a problem it is recommended to wash once more with 1 ml RNA imaging kit reaction rinse buffer after step 2.7.4.

The RNA imaging kit mentioned here represents a significant advance in RNA imaging technology in terms of ease of use and specificity and speed of reaction. This technique is primarily limited by the time it takes to label the sample. The RNA imaging kit requires a 1 hr labeling period that prevents the examination of rapid changes in global RNA that would usually occur within this timeframe. It is for this reason our first time point is restricted to 1 hr and 15 min. The technique is associated with few other limitations that largely relate to reagent compatibility with other fluorescent markers, for example, this RNA imaging kit is not compatible with phalloidin staining.

All existing approaches of studying RNA synthesis in cells are based on incorporation of modified nucleosides into the nascent RNA and the detection of incorporated labels by different means. The pioneering approach was based on using radioactively labeled RNA precursors followed by detection with autoradiography. This method led to a discovery of cell cycle stages and other important findings; however, it has a lot of disadvantages such as the cumbersome nature of radioactivity work, long exposure times and low resolution images of autoradiography^6^. The next advance in the field exploited means of immunochemistry to eliminate the necessity of radioactivity. RNA was labeled by incorporation of BrU followed by immunochemistry detection. However, efficient antibody binding required nucleic acid denaturation to improve access to DNA or RNA that caused loss of cell morphology and damaged the epitopes of many proteins, preventing their further detection with fluorescently labeled antibodies^13^. The introduction of 'click chemistry' technology simplified the detection step and the procedure compared to autoradiography and immunochemistry. The technology is based on azide-alkyne Huisgen cycloaddition reaction where terminal alkyne of 5-ethyniluridine binds with azide conjugated with the fluorophore in presence of Cu(I). The reaction is specific, rapid, does not require any additional steps, and is easily compatible with immunochemical detection of other cell constituents.

Using this RNA imaging technology we showed that cells, in the same monolayer population, have different RNA synthesis levels in response to hypoxia. We restricted our experiment to examine the effect of RNA production only; however, it is wholly possible with this RNA imaging kit to perform modified, additional multiplex reactions that allow for a more complex analysis of RNA production. For example, secondary staining using an antibody specific for markers of active transcription such as levels of phosphorylated RNA polymerase II or even components of the translation machinery to give an indication of the amount of this newly produced RNA that is converted into protein are possible. Additional proteins, such as Actin, a protein that should not change significantly with hypoxia, could be tested as an additional control. Furthermore, different cell types could be tested, as it is very likely that different cells will have different RNA synthesis rates and even different responses to hypoxia.

Use of this RNA imaging kit is quite straightforward provided the steps are performed as described. Critical steps in the protocol involve cell plating, cell fixation and staining and image acquisition. It is important to plate the cells at the described density to prevent over or under confluence at experimentation which will significantly affect the result. It is also important to use fresh fixative, ensuring the best 'snapshot' of the cell is taken and to take care when washing the cells after staining to reduce the background to a level that is acceptable for efficient analysis. Finally, the method of image acquisition is vitally important to achieve images that are of a good enough quality for subsequent analysis. We recommend using the best light microscope available to examine samples optically such as the microscope used in this protocol which is available at most research intensive centers globally.

## Disclosures

J.R.S. is Founder of Glencoe Software, Inc., an open-source US-based commercial company that contributes to OMERO.
